# Low Salivary IgA Levels Against PAc (361–386) as a Risk Factor for Root Caries in Older Adults

**DOI:** 10.1002/cre2.945

**Published:** 2024-08-05

**Authors:** Yu Ichikawa, Noboru Kaneko, Kaung Myat Thwin, Hidenobu Senpuku, Kaname Nohno, Hiroshi Ogawa

**Affiliations:** ^1^ Division of Preventive Dentistry, Faculty of Dentistry & Graduate School of Medical and Dental Sciences Niigata University Niigata Japan; ^2^ Department of Microbiology and Immunology Nihon University School of Dentistry at Matsudo Chiba Japan; ^3^ Division of Oral Science for Health Promotion, Faculty of Dentistry & Graduate School of Medical and Dental Sciences Niigata University Niigata Japan

**Keywords:** older adults, risk factor, root caries, salivary IgA antibody

## Abstract

**Objectives:**

This study aimed to assess the intricate relationship between salivary IgA antibody levels to PAc (361–386) (PPA), mutans streptococci colonization, and root caries development in older adults.

**Materials and Methods:**

This study included 307 participants aged 76 years residing in Niigata city, Japan. Clinical oral examinations were performed at baseline in 2004 and 1 year later, during which the total number of untreated and treated root caries was assessed using the root decayed, filled tooth (DFT) index. The stimulated saliva samples were collected using the spitting method during the baseline survey. Salivary IgA antibody levels to amino acid residues 361–386 of *Streptococcus mutans* PAc were quantified using an enzyme‐linked immunosorbent assay. Statistical analyses, including the *χ* ^2^ test, Mann–Whitney *U* test, and logistic regressions, were performed to examine the association of increased root DFT with the independent variables.

**Results:**

Among the 307 participants (53.1% men), the mean root DFT at baseline was 3.77 ± 3.66, and 36.5% of the study sample exhibited increased root DFT after 1 year with a mean increment of 0.36 ± 0.48. Participants with increase in root DFT after 1 year had significantly higher rates of low PPA levels (≤ 25th percentile) than those without increased root DFT (*p* = 0.020). Low PPA levels (≤ 25th percentile) were significantly more likely to have an increased risk of root caries development compared with PPA levels > 25th percentile (adjusted OR: 1.88, 95% CI: 1.09–3.25).

**Conclusion:**

Low PPA levels and root caries incidence correlated significantly, suggesting that low levels of salivary IgA antibody to PAc (361–386) may serve as a risk factor for increased root caries in older adults.

## 1 Introduction

The prevalence of dental caries and its impact on oral health among older adults has become a significant public health concern worldwide (Kassebaum et al. 2015; Chan et al. 2021). With aging, individuals often experience changes in oral health, including the development of root caries, which can cause discomfort, functional impairment, and compromised quality of life (Desai and Nair 2023). In Japan, where demographics are shifting toward an older population, understanding the dynamics of oral health and its maintenance is imperative. Previous studies conducted on the Japanese population revealed that the occurrence of root surface caries, including decayed and filled lesions, was 53.3% in community‐dwelling independently living older adults (Imazato et al. 2006) and 59.6% in older adults who required nursing care (Tokumoto et al. 2022).

As the trend of retaining more teeth increases, especially among older adults, there is a corresponding concern regarding the increase in root caries incidence (Hayes et al. 2016; Hariyani et al. 2018). Root caries can occur even in individuals with a low risk of enamel caries because the critical pH of root surface dentin for demineralization is higher than that of the enamel. The treatment of root caries is challenging, with root surface restorations often exhibiting low survival rates, emphasizing the critical importance of preventing root caries (Gil‐Montoya et al. 2014; da Mata et al. 2015). Therefore, understanding the factors and risks associated with the occurence and progression of root caries is essential for developing effective preventive strategies and promoting oral health in the aging population.

Mutans streptococci (MS), particularly *Streptococcus mutans*, are well established as primary causative bacteria implicated in the occurrence of root caries along with Lactobacilli, Bifidobacteria, and Actinomyces spp. (Loesche 1986; Takahashi and Nyvad 2011; Do et al. 2017). MS constitutes approximately one‐third of the total microbiota of cavitated dentin lesions and plays a major role in root caries progression (Shen et al. 2002). *S. mutans* contains a cell surface protein antigen referred to as PAc, which is crucial for initial attachment to tooth surfaces (Matsumoto‐Nakano 2018) and is a promising target for dental caries vaccines (Robinette et al. 2011). The antigenically significant A‐region of PAc contains amino acid residues 361–386 [PAc (361–386)], which have been identified as particularly immunogenic (Tsuha et al. 2023, 2004).

Individuals with a high salivary IgA antibody level to PAc (361–386) (PAc peptide antibody [PPA]) demonstrated significantly lower MS counts in their saliva (Tsuha et al. 2004). In addition, another study found a significantly reduced MS count in a PPA‐detected group compared with a control group in a professional oral hygiene study (Inaba et al. 2009). Furthermore, it has been observed that PPA levels correlate negatively with age (Tsuha et al. 2004). Low PPA levels correlate with higher MS counts in saliva and may precede the onset of root caries. Therefore, this study aimed to assess the intricate relationship between PPA levels, MS colonization, and root caries development in older adults with the hypothesis that low PPA levels serve as a risk factor for root caries development.

## 2 Methods

### 2.1 Study Design and Participants

The participants in this study were drawn from a Niigata cohort study focusing on Japanese older adults aged 70 years (born in 1927). In 1998, an interdisciplinary team, involving medical and dental professionals, initiated a longitudinal study among Japanese older adults to assess the relationships between general and oral health in Niigata city, Japan. Initially, 4542 individuals aged 70 years, residents of Niigata city, were sent a written request to participate in the elderly survey. Subsequently, 3695 individuals (response rate: 81.4%) were willing to participate in the study. Of these, 600 individuals were randomly selected with an equal distribution of sexes (Yoshihara, Hanada, and Miyazaki 2003; Senpuku et al. 2007). The participants provided informed consent and underwent the medical and dental examinations.

**Figure 1:**
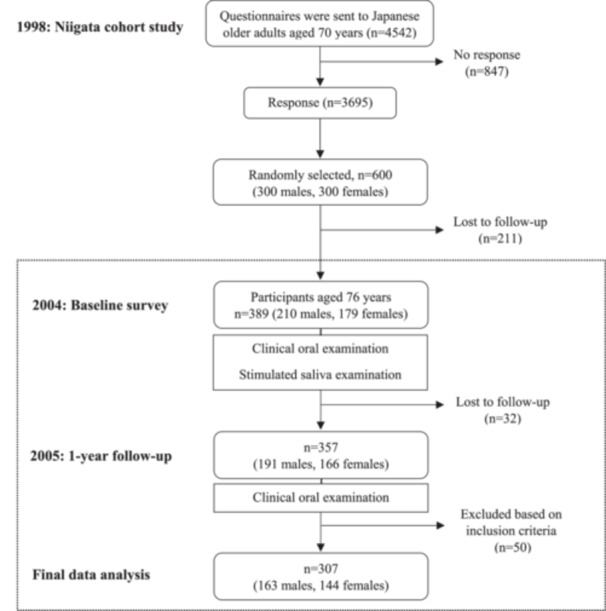
Flowchart of study protocol.

In 2004, 6 years after the Niigata cohort study began, we conducted a baseline survey, encompassing a clinical oral examination and a collection of stimulated saliva, among 389 participants aged 76 years (with 211 older adults lost to follow‐up). In 2005, clinical oral examinations were repeated for a 1‐year follow‐up survey, employing the same methodologies as in the baseline survey. Those who participated in both surveys with no missing data were included, whereas those with no root‐exposed teeth and missing data were excluded from the analysis (Figure 1). All procedures were conducted in accordance with the guidelines and regulations outlined in the Declaration of Helsinki. This study was reviewed and approved by the Ethics Committee of the Niigata University (Approval number: 12‐R1‐4‐21).

### 2.2 Clinical Oral Examination

Four calibrated dentists performed all clinical oral examinations during the baseline and follow‐up surveys. Following the WHO criteria (World Health Organization 2013), the number of present teeth, decayed teeth (DT), filled teeth (FT), and root‐exposed teeth were recorded using a plain mirror and the WHO Community Periodontal Index Probe under a portable light source. Coronal and root caries were counted separately. The total number of untreated (decayed) and treated (filled) root caries was defined as root decayed, filled tooth (DFT). The total number of teeth with caries on either coronal or root surfaces was recorded as the total DFT (crown and root).

### 2.3 Stimulated Saliva Collection

The stimulated saliva samples were collected using the spitting method, in which participants chewed paraffin wax for 3 min during the baseline survey. Subsequently, seed swabs (Culture Swab Plus, Becton Dickinson, NY, USA) were dipped in saliva for 10 s, and the samples were sent to Bio Medical Laboratory (BML, Saitama, Japan) for quantification of the number of MS. Seed swabs contain approximately 0.14 mL saliva. In preliminary experiments, the swab consistently contained 0.14 ± 0.01 mL saliva, demonstrating negligible variance. The MS count was expressed as the log CFU/swab. The remaining saliva was transported to the laboratory at 4°C and stored at −80°C until PPA measurement.

### 2.4 PPA Measurement

PPA levels were measured using an enzyme‐linked immunosorbent assay (ELISA) as previously described (Tsuha et al. 2004), with slight modifications. Briefly, a 96‐well microtiter plate (Sumitomo Bakelite, Tokyo, Japan) was coated with 100 µL of PAc (361–386) peptide (20 µg/mL) in coating buffer (pH 9.6) overnight at 4°C. Consequently, the plate was washed twice with PBS containing 0.1% Tween 20 (PBST) and blocked with 1% skim milk in PBST for 1 h at 37°C. After blocking, the plate was washed twice with PBST. As a primary antibody, 100 µL of 20‐fold diluted saliva samples was added to the wells and incubated for 30 min at 37°C. The plate was then washed four times with PBST, and 100 µL of alkaline phosphatase‐conjugated goat antihuman IgA (α chain) antiserum (Rockland, PA, USA) was added to the wells as a secondary antibody. Next, the plate was incubated for 1 h at 37°C and washed four times with PBST. Then, 100 µL of 1 mg/mL para‐nitrophenyl phosphate in diethanolamine buffer was added to the wells as a substrate. After a 1‐h incubation at 37°C, the optical density at 405 nm (OD_405_) was measured using a microplate reader (Model 680, Bio‐Rad, CA, USA). Duplicate tests were conducted, and the average of the readouts was recorded as PPA.

### 2.5 Statistical Analysis

Statistical analyses were performed using STATA 14.2 (StataCorp LLC, Texas, USA). The characteristics and general data were reported using descriptive statistics. Participants with a PPA value below the lowest quartile (≤ 25th percentile) were defined as those with low PPA levels. Increased root DFT, a dependent variable, was calculated as the difference in root DFT between the baseline and the 1‐year follow‐up surveys. Participants were then classified into two groups: those with increased root DFT and those without increased root DFT (0: not increased root DFT, 1: increased root DFT). Differences between males and females, and between the increased root DFT group and not‐increased root DFT group, were examined using the *χ* ^2^ and Mann–Whitney *U* tests. Univariable and multivariable logistic regression analyses were performed using sex, number of root‐exposed teeth, total DFT, MS count, salivary flow rate, and low PPA levels as explanatory variables to evaluate the risk of root caries development. The threshold for statistical significance for all tests was set at *p* < 0.05.

## 3 Results

Of the 600 older adults who participated in the Niigata cohort study, 389 individuals aged 76 years were included at baseline in 2004. Thirty‐two individuals did not attend the oral examination in 2005 for the 1‐year follow‐up survey. Among the remaining 357 participants, 11 individuals with no root‐exposed teeth and 39 with missing data were excluded from the analysis. Therefore, the final number of participants included in the data analysis was 307, of which 53.1% were men (*n* = 163). No significant differences were found in oral and saliva examination parameters between the older adults in the study group and those who dropped out.

Table 1 shows the baseline characteristics of the participants. The mean number of root‐exposed teeth, root DFT, and total DFT were 11.15 ± 6.89, 3.77 ± 3.66, and 12.54 ± 5.97, respectively. Significant differences were observed between sexes in root‐exposed teeth (*p* = 0.001) and root DFT (*p* < 0.001) but not in total DFT. A total of 244 participants (79.5%) had root caries, with no significant difference between male and female participants. A significant difference in salivary flow rate was observed between sexes in stimulated saliva examination (*p* < 0.001).

**Table 1:** Characteristics of participants at baseline.

Characteristics	Total (*n* = 307)	Male (*n* = 163)	Female (*n* = 144)	*p* value
Clinical oral examination				
Root‐exposed teeth	11.15 ± 6.89	12.42 ± 7.19	9.71 ± 6.26	**0.001**
Root DFT	3.77 ± 3.66	4.29 ± 3.96	3.18 ± 3.21	**< 0.001**
Participants with root caries (root DFT ≥ 1)	244 (79.5)	136 (83.4)	108 (75.0)	0.068
Total DFT (crown and root)	12.54 ± 5.97	12.51 ± 6.13	12.58 ± 5.80	0.996
Stimulated saliva examination				
MS count (log CFU/swab)	3.28 ± 1.58	3.20 ± 1.68	3.38 ± 1.46	0.599
Salivary flow rate (mL/min)	1.44 ± 0.82	1.62 ± 0.90	1.24 ± 0.69	**< 0.001**
PPA	0.48 ± 0.40	0.44 ± 0.35	0.53 ± 0.44	0.074

*Note:* Values are presented as mean ± standard deviation or number (%). Variables are analyzed by Mann‐Whitney U test or *χ* ^2^ test. Bold values indicate for statistical significance (*p* < 0.05).

Abbreviations: MS count, number of Mutans Streptococci; PPA, PAc peptide antibody; root DFT, total number of untreated (decayed) and treated (filled) root caries; Total DFT, total number of untreated (decayed) and treated (filled) coronal and root caries.

One hundred and twelve participants (36.5%) exhibited increased root DFT at the 1‐year follow‐up oral examination, with a mean increment of 0.36 ± 0.48. No sex differences were observed in the proportion or mean number of increased root DFT after 1 year. The comparisons of baseline data between participants with and without increased root DFT after 1 year are presented in Table 2. Total DFT (crown and root) at baseline was significantly higher in participants with the increased root DFT group than in those without (*p* = 0.007). Moreover, participants with increased root DFT after 1 year had a significantly higher rate of low PPA levels (≤ 25th percentile) than those without increased root DFT (*p* = 0.020).

**Table 2:** Comparisons of baseline data between participants with and without increased root DFT after 1 year (*n* = 307).

	Participants with		*p* value
	Increased root DFT (*n* = 112)	Not increased root DFT (*n* = 195)	
Sex			0.401
Male	63 (56.2)	100 (51.3)	
Female	49 (43.8)	95 (48.7)	
Clinical oral examination			
Root‐exposed teeth	11.84 ± 6.76	10.74 ± 6.95	0.173
Total DFT (crown and root)	13.75 ± 5.54	11.85 ± 6.10	**0.007**
Stimulated saliva examination			
MS count (log CFU/swab)	3.18 ± 1.63	3.34 ± 1.56	0.428
Salivary flow rate (ml/min)	1.38 ± 0.81	1.47 ± 0.83	0.429
PPA levels			0.105
1st quartile	39 (34.8)	44 (22.6)	
2nd quartile	23 (20.5)	56 (28.7)	
3rd quartile	27 (24.1)	49 (25.1)	
4th quartile	23 (20.5)	46 (23.6)	
Low PPA levels	39 (34.8)	44 (23.0)	**0.020**
(1st quartile, ≤ 25th percentile)			

*Note:* Dependent variable: participants with and without increased root DFT after 1 year. Independent variables: baseline data. Values are presented as mean ± standard deviation or number (%). Variables are analyzed by Mann–Whitney U test or *χ* ^2^ test. Bold values indicate for statistical significance (*p* < 0.05).

Abbreviations: MS count, number of Mutans Streptococci; PPA, PAc peptide antibody; root DFT, total number of untreated (decayed) and treated (filled) root caries; Total DFT, total number of untreated (decayed) and treated (filled) coronal and root caries.

Table 3 shows the results of the logistic regression analysis for increased root DFT. The analysis identified two significant predictors of increased root DFT in both unadjusted and adjusted models: total DFT (crown and root) and low PPA levels (≤ 25th percentile). Total DFT was significantly associated with increased root DFT. The crude odds ratio (OR) of total DFT was 1.08 (95% CI [95% confidence interval]: 1.03–1.13), whereas the adjusted OR was 1.06 (95% CI: 1.01–1.10) after adjusting for other factors. Low PPA levels (≤ 25th percentile) were significantly more likely to have an increased risk of root caries development compared with PPA levels > 25th percentile (OR: 1.83, 95% CI: 1.10–3.06 in the unadjusted model; OR: 1.88, 95% CI: 1.09–3.25 in the adjusted model).

**Table 3:** Logistic regression analysis for the increased root DFT.

	Unadjusted		Adjusted^a^	
	OR (95% CI)	*p‐*value	OR (95% CI)	*p‐*value
Male (*Ref:* female)	1.22 (0.77–1.95)	0.401	1.34 (0.81–2.22)	0.255
Clinical oral examination				
Root‐exposed teeth	1.02 (0.99–1.06)	0.179	1.00 (0.96–1.04)	0.903
Total DFT (crown and root)	1.08 (1.03–1.13)	**0.001**	1.06 (1.01–1.10)	**0.021**
Stimulated saliva examination				
MS count (log CFU/swab)	0.94 (0.81–1.09)	0.419	0.96 (0.83–1.12)	0.619
Salivary flow rate (mL/min)	0.87 (0.66–1.17)	0.360	0.77 (0.56–1.05)	0.100
Low PPA levels (≤ 25th percentile)	1.83 (1.10–3.06)	**0.021**	1.88 (1.09–3.25)	**0.023**
(*Ref:* > 25th percentile)				

*Note:* Bold values indicate for statistical significance (*p* < 0.05).

a Adjusted for other factors.

Abbreviations: 95% CI, 95% confidence interval; MS count, number of Mutans Streptococci; OR, odds ratios; PPA, PAc peptide antibody; root DFT, total number of untreated (decayed) and treated (filled) root caries; Total DFT, total number of untreated (decayed) and treated (filled) coronal and root caries.

## 4 Discussion

The present study investigated the association between PPA levels, MS counts, and the root caries incidence among community‐dwelling older adults aged 76 years over a 1‐year period in Niigata city, Japan. We found that the mean root DFT and root caries increment were 3.77 and 0.36, respectively. These results showed similarity with rates reported in previous systemically reviewed studies (Griffin et al. 2004; Hariyani et al. 2018). Furthermore, our findings elucidated the risk factors and predictors of root caries development. Specifically, a significant association was observed between PPA levels and an increased root caries incidence after 1 year. Participants with low PPA levels (≤ 25th percentile) exhibited a nearly twofold higher risk of experiencing increased root caries than those with higher PPA levels. Although PPA levels exhibited a strong correlation with IgA levels against skim milk as an analog of total IgA levels, the analysis revealed that substituting IgA antibody level against skim milk for PPA did not demonstrate a significant relationship with the increase in root caries. These findings may be attributed to the crucial role of salivary IgA antibodies in the oral immune defense system, as they bind to and neutralize cariogenic bacteria (Wu et al. 2020). Moreover, salivary IgA antibodies can interfere with the adherence and colonization of cariogenic bacteria to dental surfaces (Marcotte and Lavoie 1998; Wu et al. 2020). Therefore, lower PPA levels may decrease inhibition of bacterial attachment to root surfaces, thereby promoting bacterial colonization and subsequent root caries development. Our findings suggest the potential importance of PPA as a valuable biomarker for identifying individuals with increased risk of root caries, thus enabling targeted preventive interventions.

Our study also identified total DFT (crown and root) as a significant predictor of increased root caries incidence over the study period. This finding aligns with previous studies which indicated that factors including root caries, root DFT (Sánchez‐García et al. 2011), coronal caries (Scheinin et al. 1994; Hayes et al. 2016), and untreated root caries (Takano et al. 2003) serve as significant risk factors for the root caries development. Understanding the cumulative impact of caries experience highlights the importance of comprehensive caries management strategies that address both coronal and root surfaces.

Our study did not find a significant association between MS counts and increased root caries incidence, despite MS being commonly implicated as the primary causative bacteria in dental caries. This suggests that the microbial dynamics of root caries may differ from those observed in coronal caries, where MS counts serve as reliable indicators of caries risk. Root caries, like dental caries, initiates with tooth surface demineralization due to low pH bacteria, including MS (Takahashi and Nyvad 2016). However, root caries follows a more complicated course involving various bacteria, not limited to acidogenic bacteria but also including proteolytic bacteria (Loesche 1986; Do et al. 2017). Compared to coronal caries, the association of MS with root caries has been reported to be lower (Scheinin et al. 1994), potentially explaining the absence of a significant relationship between MS counts and root caries incidence in our findings. Further research is needed to elucidate the complexities of microbial composition and their roles in the progression of root caries.

This study had some limitations. First, we measured the total number of MS counts, including *S. mutans* and *S. sobrinus*, which may have slightly influenced the assessment of the relationship between PPA and MS. Although *S. sobrinus* possesses a surface protein antigen similar to PAc, known as PAg, the distinction between PAg and PAc may have subtle effects on our analysis. Nevertheless, both antigens share certain amino acid residues, YEAL‐L‐QY, as a common epitope (Okahashi et al. 1993), thereby minimizing the potential impact of this difference on our findings. Second, the exclusion of individuals with no root‐exposed teeth and missing data may have caused selection bias, potentially affecting the generalizability of the results. Third, the measurement of MS counts and PPA levels may be subject to variability inherent to laboratory assays, necessitating further validation in larger cohorts. Finally, unexpected situations, including delayed researcher agreement and responsibilities, resource constraints, and the COVID‐19 pandemic, significantly prolonged the completion of this study's report. Despite these challenges, our study contributes valuable insights into the risk factors and predictors of root caries development among older adults, highlighting the importance of targeted preventive interventions and personalized oral health care strategies.

## 5 Conclusion

Our study revealed an association between low levels of salivary IgA antibody to PAc (361–386) and root caries occurrence, emphasizing the importance of identifying low PPA levels as a significant predictor of increased root caries in older adults. This finding suggests the importance of incorporating salivary biomarkers into root caries assessment and preventive strategies. Future longitudinal research with a larger population should focus on elucidating the mechanisms driving root caries progression and validating the utility of salivary biomarkers in clinical practice.

## Author Contributions

Conceptualization and study design: Noboru Kaneko, Hidenobu Senpuku, and Hiroshi Ogawa. Data collection: Noboru Kaneko, Kaname Nohno, and Hiroshi Ogawa. Data cleaning and verification: Yu Ichikawa and Noboru Kaneko. Data analysis and interpretation: Yu Ichikawa, Noboru Kaneko, and Kaung Myat Thwin. Drafting the initial manuscript: Yu Ichikawa and Kaung Myat Thwin. Critically revision of the manuscript: Noboru Kaneko and Kaung Myat Thwin. Approval of the final manuscript: All authors.

## Ethics Statement

This study was reviewed and approved by the Ethics Committee of the Niigata University, Japan (approval number: 12‐R1‐4‐21).

## Consent

The participants who agreed to participate in the study signed their written consent forms. All procedures were conducted in accordance with the guidelines and regulations outlined in the Declaration of Helsinki.

## Conflicts of Interest

The authors declare no conflicts of interest.

## Data Availability

The data sets are not publicly available due to the restrictions by the Ethics Committee of Niigata University, Japan, to protect the participants' privacy.
